# Optical Trapping and Manipulating with a Silica Microring Resonator in a Self-Locked Scheme

**DOI:** 10.3390/mi11020202

**Published:** 2020-02-15

**Authors:** Victor W. L. Ho, Yao Chang, Yang Liu, Chi Zhang, Yuhua Li, Roy R. Davidson, Brent E. Little, Guanghui Wang, Sai T. Chu

**Affiliations:** 1 Department of Physics, City University of Hong Kong, Kowloon 999077, Hong Kong, China; wailokho2-c@my.cityu.edu.hk (V.W.L.H.); yuhuali3-c@my.cityu.edu.hk (Y.L.); 2 College of Engineering and Applied Sciences, Nanjing University, Nanjing 210093, China; 131270047cy@sina.com (Y.C.); MF1934012@smail.nju.edu.cn (Y.L.); MF1834026@smail.nju.edu.cn (C.Z.); 3 QXP Technology, Xi’an 710311, China; roy.davidson@qxptech.com; 4 State Key Laboratory of Transient Optics and Photonic, Xi’an Institute of Optics and Precision Mechanics, Chinese Academy of Sciences, Xi’an 710119, China; brent.little@opt.ac.cn

**Keywords:** optical trapping, microring resonator, micro manipulation

## Abstract

Based on the gradient force of evanescent waves in silica waveguides and add-drop micro-ring resonators, the optical trapping and manipulation of micro size particles is demonstrated in a self-locked scheme that maintains the on-resonance system even if there is a change in the ambient temperature or environment. The proposed configuration allows the trapping of particles in the high Q resonator without the need for a precise wavelength adjustment of the input signal. On the one hand, a silicon dioxide waveguide having a lower refractive index and relatively larger dimensions facilitates the coupling of the laser with a single-mode fiber. Furthermore, the experimental design of the self-locked scheme reduces the sensitivity of the ring to the environment. This combination can trap the micro size particles with a high stability while manipulating them with high accuracy.

## 1. Introduction

Optical tweezers are widely used for capturing micro-particles or cells, due to the strong gradient force of highly focused laser beams [1,2]. To improve the level of system integration and trapping stiffness, researchers have explored near-field optical forces of evanescent fields around photonic waveguides in recent years [3,4,5,6,7]. Several methods for particles trapping and manipulation have been demonstrated, such as the optofluidic switching of nanoparticles [8,9], nanoparticle sorting [10,11], storing of nanoparticles [12,13], and precise manipulation in nanophotonic Standing Wave Array Traps (nSWATs) [14,15,16], among which the micro-ring resonator plays an important role. Various demonstrations of optical trapping with planar microring resonators have previously been reported, e.g., the authors of [17] studied particles that are trapped and manipulated by adjusting the input tunable lasers both on and off resonance. A resonator is an energy storage system with a feedback system where the optical intensity in the cavity is enhanced when it is on resonance. Compared to the waveguide trapping configurations, resonant cavity trapping presents two advantages [17]; the strong force enhancement due to the high field confinement in a cavity, and the cavity perturbation induced by the trapped object, which could serve as a highly sensitive probe for analyzing the physical properties of the objects [18,19]. The trapping and manipulation of micro particles on planar ring resonators have recently been demonstrated using silicon photonic crystal resonators [20]. However, to be able to trap the particles continually without interruption, it is vitally important to keep the input laser frequency aligned with the resonance frequency of the resonator, as the resonant frequency may shift during the operation from any changes in the ambient temperature and environment. The misalignment of the laser with the resonance frequencies reduces the intracavity power of the resonator, causing the particle to dislodge from the trap, resulting in the loss of control of the trapped particle. [21]. This is especially important when applying optical trapping with high-Q ring resonators in highly precise measurements [17] and in biomedical [22,23], biochemical, and chemical sensing [24]. To improve the stability of the trapping process, we proposed a self-locked scheme which allows the signal to remain on resonance regardless of the variation in the ambient temperature and environment [25]. 

**Figure 1:**
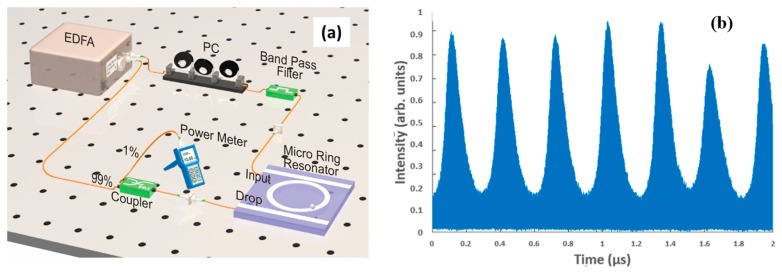
(**a**) The optical trapping system with a highly-doped silica glass ring resonator in a self-locked configuration, EDFA is an erbium-doped fiber amplifier, and PC is a three-ring polarization controller. (**b**) The fast detector output from the tap showing Q-switching in the fiber loop cavity. The repetition rate of the Q-switched pulses is approximately 100 kHz with the high frequency component within the Q-switched pulses at 6.6 MHz, which corresponds to a fiber loop length of 30 m.

The proposed self-locked configuration is shown in Figure 1a, where we replace the tunable laser in the conventional open-loop configuration with a bandpass filter and arrange the circuit in a closed-loop configuration. The configuration is similar to the passive mode-locked or fiber laser configuration where the external cavity acts as the gain cavity and the combination of the bandpass filter with the ring resonator acts as the wavelength selective element [21]. Since the external cavity has a much finer free-spectral range (FSR) spacing and narrower linewidth than the ring resonator, the system remains in the lasing condition regardless of any resonance frequency shift encountered by the ring resonator. Additional control elements, such as switches, can be added to the basic configuration in Figure 1a to select the propagation direction of the particles, while the propagation speed can be adjusted from the power of the amplifier. When it is on resonance, the intracavity power in the microring resonator is proportional to its Q factor, with *Q* = *λ*/Δ*λ*, where Δ*λ* is the full-width half maximum (FWHM) of the resonance peak. As the light resonates and builds up within the resonator, the intracavity power and field in the microring resonator can be enhanced by orders of magnitude [25] above the input power. Thus, the field in the ring can be much higher than the bus waveguide, and trapping can be achieved with a much lower input power as compared to particle trapping with a simple waveguide. In this work, we demonstrate that the proposed scheme can trap polystyrene (PS) beads of sizes up to 3 µm with an average input power as low as a few tens of mW.

## 2. Materials and Methods 

The self-locked optical trapping setup used in the experiment in Figure 1a consists of an amplifying fiber ring loop of approximately 30 m [21] corresponding to an FSR of approximately 6.6 MHz. The FSR of the external cavity loop represents the resolution of the measurement system, while the role of the ring resonator is to select which of the resonance frequencies of the external loop will be amplified. By choosing the bandpass filter to be larger than the FSR of the ring resonator, one ensures that at least one of the ring resonances is excited regardless of any resonance frequency drift. However, to prevent mode hopping between different resonances, the bandpass filter is selected to be close to the FSR of the ring resonator. Since the fiber loop of the main cavity is relatively long, one does not expect continuous wave (CW) lasing from the configuration due to modulation instability. To investigate the dynamic behavior of the optical signal in the fiber loop, we measured the time response of the optical signal at the tap with a high-speed optical detector. The output waveform from the detector is shown in Figure 1b, which shows an irregular pattern of Q switch pulses due to the dynamic instability in the long cavity loop. 

The investigation of the trapping capability with integrated optical waveguides and with the enhancement of the light intensity in ring resonators has been demonstrated in silicon-rich integrated optical circuits, such as silicon nitride waveguides [26]. The ring resonator add/drop filter used in this experiment is fabricated with a low-loss highly doped glass with a core index of 1.70, a cladding index of 1.445, and with a waveguide cross section of 1.45 µm × 1.45 µm to allow a higher field penetration into the cladding for trapping while maintaining a reasonable Q factor. With the ring radius at 47 µm, it corresponds to an FSR of 569 GHz. The gap separation between the ring and the bus waveguide is 0.85 µm, so that the FWHM of the resonance peak is 21 pm at around 1550 nm. This corresponds to a Q factor of approximately 75,000 when the device is measured in air but the FWHM, Q factor, and FSR change to 27 pm, 65,000 and 572 GHz, respectively, when it is immersed in water. It is important to note that the trapping force is a function of the intensity enhancement factor B of the resonator, which measures the amount of amplification of the input in the resonator cavity. When the device is placed in air the resonator enhances the intensity by approximately 50 times, but this is reduced to only 30 times when it is in water due to the weaker field confinement. Furthermore, to provide a flat surface for the trapping application, the device was planarized to the top of the waveguide. The device was pigtailed to an optical fiber array with a coupling loss of approximately 5 dB/facet. In the experiment, we first deposited deionized water droplets on the surface of the chip and placed the PS particles in the vicinity of the waveguide structures. A thin cover glass was then placed on the water surface of the chip to provide an even distribution of the water solution across the surface and to allow the experiment to be monitored via a microscope and Charge Coupled Devices (CCD) camera system. 

## 3. Simulation

**Figure 2:**
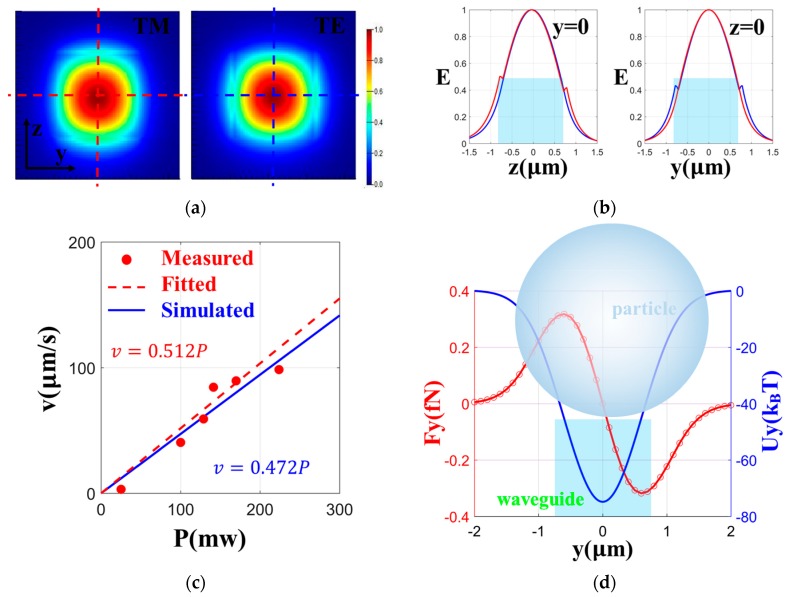
(**a**) The TM and TE mode distribution in the waveguide cross section. (**b**) The electric field *E* of four dotted lines passing through the centre of the section with a change with the z-direction (left) or y-direction (right). The line in TM is blue, and the line in TE is red. (**c**) The fitting red dotted line between the particle velocity *v* and power *P* obtained by the data in the experiment (red dots). The corresponding simulation results are represented with a blue line. (**d**) The gradient force and the corresponding optical potential well along the y-direction.

To investigate the interaction between the light field on the waveguide and the particle, we calculated the distribution of the light field in both the Transverse Magnetic (TM) mode and Transverse Electric (TE) mode of the waveguide cross section. The light transmission is in the x-direction, the vertical direction of the cross section is the z-direction, and the horizontal direction is the y-direction. As shown in Figure 2a,b, the evanescent field in the TM mode was stronger in the z-direction, and that in the TE mode was stronger in the y-direction. Based on the requirement of trapping particles at the waveguide top surface, the TM mode was adopted for subsequent simulations. The simulations were carried out using the commercial software packages from Lumerical, such as FDTD and MODE Solutions, with the finite-difference time-domain algorithm to solve the electromagnetic field distribution, while the volumetric technique algorithm was used to calculate the optical force on the particles. The volumetric technique algorithm uses the Coulomb force formula, the Lorentz formula, and the Maxwell’s equations to solve the optical force at a point on a particle, and the optical force on each point is integrated over the entire particle volume to obtain the optical force on the particle. Compared with a silicon waveguide, our cross-section mode was larger and had the advantage of attaching an optical fiber, which allowed the device to be portable for the ease of the setup. Both the TM and TE mode had relatively small losses. It can be seen from the simulation that the TM mode played a major role in capturing particles on the upper surface of the waveguide.

We also simulated the scattering force along the x-direction and the gradient force along the y-direction on a particle with a diameter of 3 µm. The scattering force was stable at around 0.01 pN/mW, which formed the driving force for the particles to advance along in the x-direction. The particle velocity of the simulation was calculated by Stokes’ drag formula:

*v = F/3πDη* (1)

where *Fs* is the scattering force, *D* is the particle diameter, and *η* is the viscosity coefficient.

As shown in Figure 2c, the simulation and experimental results were in good agreement within a certain power range. When the gradient force was integrated along the y-direction displacement, the corresponding optical potential well could be obtained. In the case of 20 mW, the depth of the potential well in Figure 2d was around 80 k_B_T, when the polystyrene particles with a diameter of 3 µm were used at a speed of 100 µm/s, according to the formula of centrifugal force:

(2) $$F=mv^{2}/r$$

where *F* is the centrifugal force, *m* is the mass of the particle, and *v* is the viscosity. The required centripetal force is about 0.1 fN. In the simulation, when the incident light power was 20 mW, the maximum gradient force on the particle was 0.3 fN, so the gradient force could provide the centripetal force required for the particle to move around the ring.

**Figure 3:**
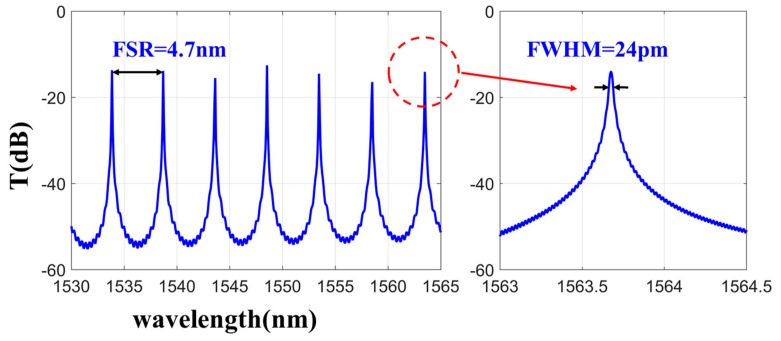
The simulated transmission spectra of rings at the drop from 1530 nm to 1565 nm.

We also simulated the light field of the whole structure and plotted the transmission spectra of the rings at the drop port, as shown in Figure 3. It was determined to have an FSR of 4.7 nm with an FWHM of 24 pm at around 1564 nm, with a Q factor of 65,154. These results are consistent with the experimental results shown in the next section. The calculated Q factor at the resonance wavelength (*λ*) was compared with the experiment to verify the simulation.

**Figure 4:**
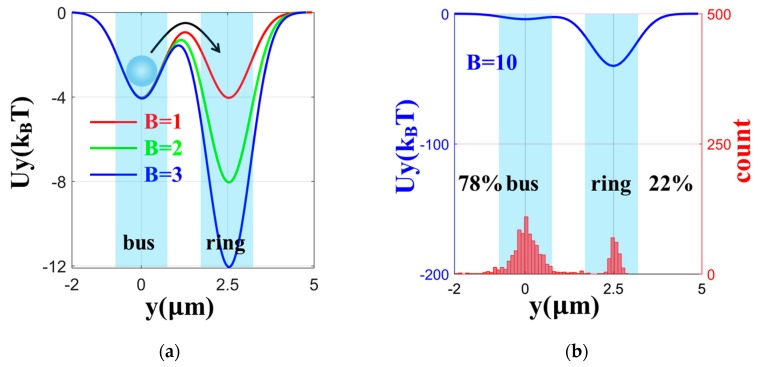

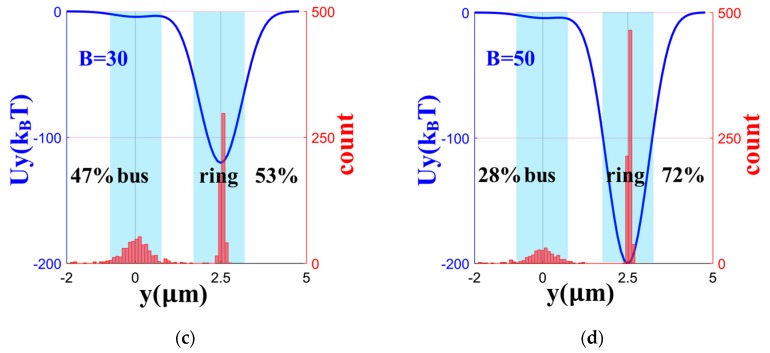
(**a**) Potential wells of 3 µm diameter spheres in the y-direction with B = 1, 2, and 3. (**b**) The particle distribution on the ring and bus in 5 s when B = 10. The particle distribution on the ring and bus in 5 s when (**c**) B = 30 and (**d**) B = 50.

Using the different enhancement factors B, we calculated a series of potential wells along the y-direction, which reflected the process of particle transfer from bus waveguide to ring. In order to show the process better, we calculated the particle motion considering the Brownian motion. The relationship between the displacement of the particle and time was obtained by solving the Newton equation of motion for a single particle, including the optical gradient force, the viscous resistance, and the Brownian force [27]. According to the displacement statistics of 1000 particles in 5 s, the distribution ratio of particles on the bus waveguide and ring was obtained, as shown in Figure 4. The simulation time was set to 5 s, because the gap between the ring and the bus waveguide changes little during this time, taking into account the particle actual x-direction velocity [12]. With the increase of B, the proportion of particles jumping from the bus to ring increased, as shown in Figure 4b–d. This is a good explanation for the phenomenon of particles transferring from bus waveguide to ring.

## 4. Results and Discussion

Q-switched pulses with a high frequency component of ~6.6 MHz equal to the free spectral range of the external cavity loop similar to those shown in Figure 1b were observed in this experiment. Stable periodic pulses can also be observed when the external cavity length matches integer multiples of the microring resonator cavity length, but due to the long cavity length stable passive mode locking is not sustainable. Methods such as adding modulation or drastically reducing the length of the external loop will be needed to maintain stable mode locking. Nevertheless, the self-locked configuration allows the microring resonator to always remain on-resonance, producing the maximum trapping force on the resonator. 

**Figure 5:**
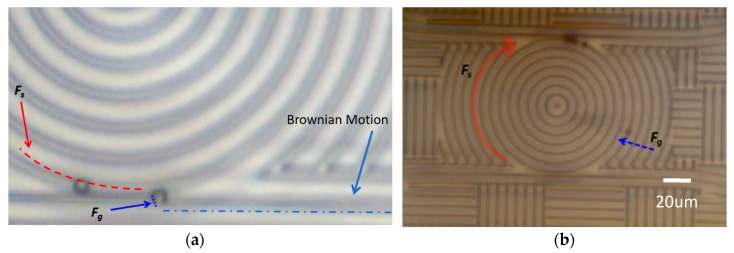
(**a**) The optical image of the ring resonator (highlighted by the red dotted line with the ring and the blue dotted line with the bus) prior to the EDFA being switched on. This figure shows two beads delivered along the bus waveguide to the ring. The average speed of a bead on the bus and the ring is 3.13 μm/s and 40.38 μm/s, and the electrical field of the ring is stronger than the bus waveguide. (**b**) The demonstration of the two trapping forces produced by the intensity in the waveguide. The four beads are being pushed around the ring resonator by the scattering force F_s_ indicated by the red arrow, and the single bead outside the ring is being pulled toward the waveguide by the gradient force F_g_, indicated by the blue arrow.

In the experiment, the particles and deionized water solution were first dropped onto the top surface of the chip. Since there is no fluidic cell to guide the particles, and in view of the flat chip surface, the particles flowed freely on the chip surface when the EDFA was off. When the power of the amplifier was switched on, power was delivered to the bus waveguide, and the intensity in the ring resonator started to build up. At this point, the particles that were in the vicinity of the waveguides started to migrate toward the waveguides due to the gradient force produced by the intensity in the waveguide. When the particles were close enough to the waveguide, they would be trapped by the waveguides and pushed along the waveguide by the scattering force, as shown in Figure 5a. For those particles trapped by the input waveguides, they would be pushed along the waveguide until they reached the ring resonator where they could be attracted and trapped by the ring resonator because of the higher intensity inside the ring cavity. 

Since the intensities in the bus and ring waveguides are not the same, the particle encounters different scattering forces when it is on the bus and on the ring waveguides. The ratio between the speeds of the particle when it is on the bus waveguide and when it is on the ring resonator provides a direct measure of the intensity enhancement factor of the ring resonator. Figure 5a and Video S1 show two beads that were initially traveling along the bus waveguide and were later trapped by the ring resonator when they arrived at the coupling region between the ring and the bus. From the two measured speeds, we found that the speed of the particle was approximately 15 times faster when it was on the ring resonator, which corresponded to the intensity differences between the ring and the bus. Figure 5b and Video S2 shows the two different force observed in the experiment: the scattering force (Fs) that pushed the group of beads along the ring resonator in the direction indicated by the red arrow, and the gradient force (Fg) that pulled the free-floating particle toward the waveguide indicated by the bluearrow. 

**Figure 6:**
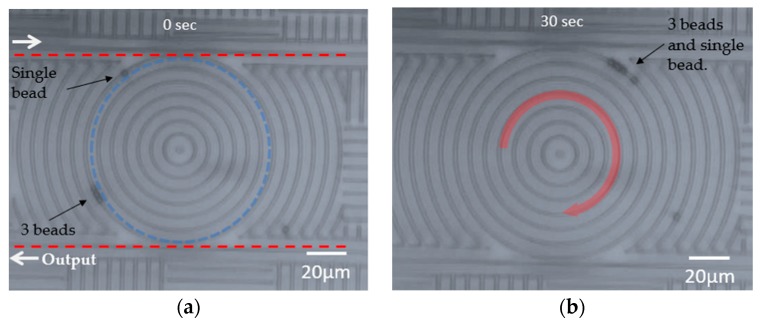
Four particles trapped on top of the ring waveguide in two groups traveling at different speeds. (**a**) Initially, they were separated by approximately one quarter of the ring circumference, with the group of three beads traveling at a higher speed. (**b**) The group of three beads caught up with the single bead after 30 s.

We also observed a series of three particles that were trapped and pushed along the ring resonator at the same time while a single particle was trapped by the same ring, as shown in Figure 6a. In the example shown in Figure 6, the four particles were initially separated into two groups, a single particle and a group of three particles. The two groups of particles travelled at different speeds, with the group of three travelling at a higher speed than the single particle. The measured speed of the single particle was 19.7 μm/s, while the speed of the peloton of three particles was 23.6 μm/s. Figure 6a,b are still images of the Video S3 taken at 30 s apart. Initially, the single particle was approximately one quarter of the ring circumference ahead of the peloton, but at 30 s the peloton caught up with the single particle due to the reduced aerodynamic drag [28]. It is also interesting to note that all three particles received the same amount of force due to the same field overlap they received, but they shared the burden to overcome the drag.

**Figure 7:**
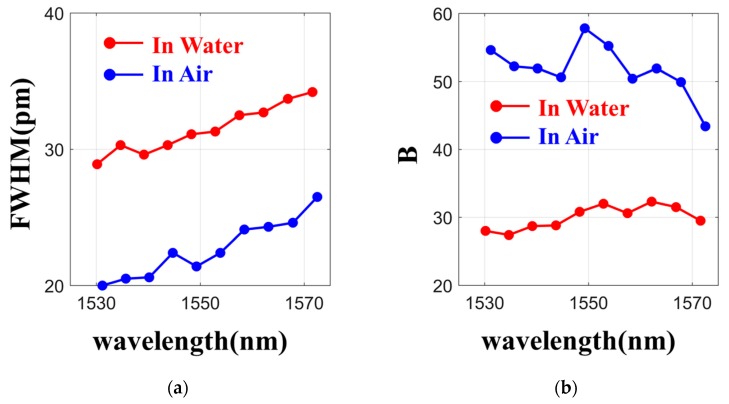
(**a**) The FWHM in different wavelengths in air and water. (**b**) The confinement factor (B) in different wavelengths in air and in water.

The speed of the particle can also be adjusted by controlling the power of the EDFA; however, the amplitude and repetition rate of the Q-switched pulse are highly sensitive to changes in the polarization and cavity length variation. Hence, we can only relate the average power in the resonator to the trapping speed, instead of the peak pulse power. Table 1 shows the resonance wavelength and measured speed of the 3 μm size PS particle at the different average powers with the particle on the waveguide and on the resonator. At the average input power of 14.1 dBm, the speed of the particle was measured at 3 μm/s, however the speed increased to 40.4 μm/s when it was transferred onto the ring resonator. Here, one can roughly adjust the speed of the particle on the ring resonator from 40.4 μm/s to 98 μm/s by changing the input average power from 14.1 dBm to 17.5 dBm. Figure 7a shows the measured FWHM of the resonance peak as a function of the wavelength when the device is in air and when it is immersed in water. For the ring resonator used in the experiment, the FWHM of the resonance peak increased by approximately 50% when the device was immersed in water as compared to when it was in the air. Besides increasing the FWHM, the higher refractive index and absorption of water also decreased the field enhancement factor B, as shown in Figure 7b. 

Finally, in the experiments described in this section, the device was exposed to the ambient environment without any mechanism to control the device temperature or resonance location; therefore, the resonance frequency was allowed to drift. Although the resonance frequency can drift on the order of tens of pm due to the intensity induced temperature change, as shown in Table 1, the self-locked scheme was able to keep the system on resonance, maintaining the trapping capability at all times.

## 5. Conclusions

We have proposed and demonstrated an optical trapping scheme that allows the continuous trapping and manipulation of polystyrene beads, with sizes as large as 3 µm in diameter, on an integrated optical microring resonator. The proposed scheme employs a closed-loop configuration that can keep the microring resonator on-resonance at all times, trapping the particles on top of the resonator regardless of how the particles affect the resonance frequency of the resonator. Compared to the conventional trapping configuration, the proposed system eliminates the need for the *in-situ* adjustment of an input wavelength to align with the ring resonance. We have carried out a detailed simulation of the trapping process that generally agrees with the experimental results. We have shown that the speed of the particles can be adjusted by the EDFA power level, where the particle can be propelled around the ring resonator with speeds close to 100 µm/s. It is important to point out that the signal that travels around the resonator is in the form of pulses, where each of the pulses contains the instantaneous time response of the environment of the resonator. One can explore the characteristics of these pulses to provide real-time information of the particles on the resonator in highly sensitive sensing systems. 

## Figures and Tables

**Table 1:** The resonance wavelength and estimated average power in the waveguide and the resonator with the relative trapping speed.

**Resonance Wavelength (nm)**	**Average Power in the Input Waveguide (dBm)**	**Average Power in the Resonator (dBm)**	**Location of the Particle**	**Trapping Speed (** **μm/s)**
1555.35	14.1	N/A	Bus	3.13
1555.35	14.1	20.0	Ring	40.4
1555.38	15.1	21.1	Ring	59.1
1555.39	15.5	21.5	Ring	84.4
1555.40	16.3	22.3	Ring	89.5
1555.41	17.5	23.5	Ring	98.4

## Supplementary Materials

The following are available online at https://www.mdpi.com/2072-666X/11/2/202/s1, Video S1: Refer to Figure 5a, Video S2: Refer to Figure 5b, Video S3: Refer to Figure 6a,b.
